# Gender specific complications in female diabetic patients: an integrative view

**DOI:** 10.1186/1878-5085-5-S1-A78

**Published:** 2014-02-11

**Authors:** Olga Golubnitschaja

**Affiliations:** 1 Molecular Diagnostics, Radiological Clinic, Medical Faculty, Friedrich-Wilhelms-University of Bonn, Germany

## Objective

Current diabetes care is considered as insufficient regarding early diagnostics, targeted prevention and treatments tailored to the person. Further, gender specific diagnostics and treatment are not the issue of currently applied medical services in diabetes care. This is to understand status quo in diabetes complications and co-morbidities more typical for the female sub-populations.

## Material and methods

Pubmed literature search within the years 1980-2013 [1].

## Results and interpretation

Cardiovascular disease (CVD) is the best acknowledged complication in patients with Diabetes mellitus type 1 and type 2. It has been well documented that female diabetics demonstrate poorer outcomes of CVD. A combination of the female gender and diabetes is the best recognised risk factor for high operation mortality by and low efficacy of the replacement of calcified valves [2]. Further, cardiovascular diseased women, in particular female diabetics, have poorer prognosis of oncologic diseases. The most frequent oncologic complications and co-morbidities in female diabetics are the following ones [3,4]:

❖ endomentrium carcinoma (4.8-times higher prevalence in type 1 diabetes and 2.2-times higher in diabetes generally)

❖ ovarian carcinoma (2.42-times higher risk in female diabetics *versus* general female subpopulation)

❖ liver cancer (2.0-times higher risk)

❖ lymphoma (1.9-times higher risk)

❖ uterus carcinoma (1.7-times higher risk)

❖ rectum cancer (1.7-times higher risk)

❖ stomach cancer (1.6-times higher risk)

❖ leukaemia (1.4-times higher risk)

❖ kidney cancer (1.4-times higher risk)

❖ pancreatic cancer (1.3-times higher risk)

❖ breast cancer (1.2-times higher risk)

❖ lung cancer (1.1-times higher risk).

## Conclusions

Common risk factors moderating the outcomes in the most frequent female pathologies, namely DM type 2, CVD and breast cancer are progressing age, overweight, poor diet, physical inactivity and depression. Modifiable risk factors persist from childhood and adolescence into adulthood and tend to cluster with synergistic negative effects for consequent manifestation of co-morbid pathologies [5]. However, there are significant knowledge deficits concerning “typical” *versus* “atypical” co-morbidity profiles in diabetic females.

## Recommendations

Although the coincidence is common in women, consistent data do not exist to recognise complications and co-morbid pathologies by patient profiling. Complications and complex clinical situations in elderly populations should be considered as the persistent challenge that requires new strategies in healthcare. Integrative medical approaches are strongly desirable to analyse common risk factors as well as their individual and synergistic effects. Frequent *versus* rare complication profiles in cardiovascular diseased patient cohorts should be created for advanced treatment regiments.
